# METTL14 aggravates podocyte injury and glomerulopathy progression through N^6^-methyladenosine-dependent downregulating of Sirt1

**DOI:** 10.1038/s41419-021-04156-y

**Published:** 2021-09-27

**Authors:** Zhihui Lu, Hong Liu, Nana Song, Yiran Liang, Jiaming Zhu, Jing Chen, Yichun Ning, Jiachang Hu, Yi Fang, Jie Teng, Jianzhou Zou, Yan Dai, Xiaoqiang Ding

**Affiliations:** 1 Division of Nephrology, Zhongshan Hospital, Fudan University, Shanghai, PR China; 2 Shanghai Medical Center of Kidney, Shanghai, PR China; 3 Shanghai Institute of Kidney and Dialysis, Shanghai, PR China; 4 Shanghai Key Laboratory of Kidney and Blood Purification, Shanghai, China; 5 Hemodialysis Quality Control Center of Shanghai, Shanghai, China

**Keywords:** Cell death, Glomerular diseases

## Abstract

Podocytes are known to play a determining role in the progression of proteinuric kidney disease. N^6^-methyladenosine (m6A), as the most abundant chemical modification in eukaryotic mRNA, has been reported to participate in various pathological processes. However, its role in podocyte injury remains unclear. In this study, we observed the elevated m6A RNA levels and the most upregulated METTL14 expression in kidneys of mice with adriamycin (ADR) and diabetic nephropathy. METTL14 was also evidently increased in renal biopsy samples from patients with focal segmental glomerulosclerosis (FSGS) and diabetic nephropathy and in cultured human podocytes with ADR or advanced glycation end product (AGE) treatment in vitro. Functionally, we generated mice with podocyte-specific METTL14 deletion, and identified METTL14 knockout in podocytes improved glomerular function and alleviated podocyte injury, characterized by activation of autophagy and inhibition of apoptosis and inflammation, in mice with ADR nephropathy. Similar to the results in vivo, knockdown of METTL14 facilitated autophagy and alleviated apoptosis and inflammation in podocytes under ADR or AGE condition in vitro. Mechanically, we identified METTL14 knockdown upregulated the level of Sirt1, a well-known protective deacetylase in proteinuric kidney diseases, in podocytes with ADR or AGE treatment. The results of MeRIP-qPCR and dual-luciferase reporter assay indicated METTL14 promoted Sirt1 mRNA m6A modification and degradation in injured podocytes. Our findings suggest METTL14-dependent RNA m6A modification contributes to podocyte injury through posttranscriptional regulation of Sirt1 mRNA, which provide a potential approach for the diagnosis and treatment of podocytopathies.

## Introduction

The terminally differentiated podocytes, which are indispensable components of glomerular filtration barrier, play a critical role in the pathomechanism of proteinuric kidney disease, such as focal segmental glomerulosclerosis (FSGS) and diabetic nephropathy (DN) [1, 2]. A variety of genetic and environmental damage factors can target podocyte and lead to foot process effacement and eventual detachment, thereby resulting in proteinuria and progression to glomerulosclerosis [3]. The lack of effective interventions on preventing podocyte injury demands a better understanding of the key and universal molecules involved in various podocytopathies, which may provide potential diagnostic and therapeutic measures for patients with proteinuric kidney disease [4].

N^6^-methyladenosine (m6A) methylation, as the most prevalent internal modification in eukaryotic mRNA, has attracted increasing attention in recent years [5]. Catalyzed by the m6A methyltransferases (“writers”), a methyl group is dynamically installed to the N^6^ site of adenine within the consensus sequence of RRACH, which is preferentially enriched around the stop codon, 3′‐untranslated regions (3′‐UTRs) and the long internal exons in RNAs [6]. Methyltransferase-like 3 (METTL3), methyltransferase-like 14 (METTL14), and Wilms tumor 1-associated protein (WTAP) are reported to be the core components of this writer complex [7, 8]. The reversible process is mediated by the m6A demethyltransferases (“erasers”), such as fat mass and obesity‐associated protein (FTO) [9] and alkB homolog 5 (ALKBH5) [10]. Recognized by “reader” proteins, the posttranscriptional RNA m6A modification modulates RNA stability and export, transcription splicing, protein translation and other cellular processes [11]. Recent studies demonstrate that dysregulated RNA m6A modification is involved in various pathological processes, such as abnormal cell differentiation and pluripotency [12], brain diseases [13] and tumorigenesis [14, 15]. Particularly, METTL14 is reported to be closely associated with kidney diseases, such as renal ischemic reperfusion injury and vascular calcification induced by uremic toxins [16, 17]. However, the role of RNA m6A methylation and its regulatory mechanism remain unclear in podocytopathies.

To better understand the effect of m6A modification in podocytopathies, we first measured the m6A RNA levels and the expression pattern of m6A modifying enzymes in adriamycin (ADR) and DN, and found METTL14-mediated evident upregulation of RNA m6A methylation in the two proteinuric kidney disease models. Podocyte-specific deletion of METTL14 ameliorated podocyte injury and glomerulosclerosis in ADR-treated mice. Furthermore, we observed that genetic ablation of METTL14 in vitro promoted autophagy and attenuated apoptosis and inflammation in podocytes with ADR or advanced glycation end product (AGE) treatment. These results indicate the elevated mRNA m6A methylation mediated by METTL14 is responsible for the progression of podocytopathies.

Sirt1, as the most studied member of the sirtuin family belong to class III NAD^+^-dependent histone deacetylases (HDACs), has been reported to deacetylate the targeted transcription factors and regulate a variety of cellular functions, including autophagy, apoptosis, inflammation, mitochondrial biogenesis and metabolism homeostasis [18, 19]. Studies in kidney diseases have found that Sirt1 is abundant in renal tissues and contributes to reduce acute kidney injury, alleviate renal fibrosis and delay renal aging [20]. The protective effect of Sirt1 in podocytopathies has also attracted extensive attention. Evident reduced Sirt1 expression is observed in human and animal models with podocytopathies [18]. Sirt1 agonists such as resveratrol can alleviate glomerular injury in proteinuric kidney disease [21], while podocyte-specific deletion of Sirt1 aggravates proteinuria and podocyte injury in mice with ADR or DN [22, 23]. Apart from promoting deacetylation of downstream transcription factors, including p53, FOXO, STAT3 and RelA/p65NF-kB, Sirt1 can maintain actin cytoskeleton integrity and prevent podocyte injury through deacetylating cortactin [24]. However, the regulatory mechanisms of Sirt1 downregulation in podocytopathies remain incompletely understood. Mechanistically, we found METTL14 negatively regulated the stability of Sirt1 mRNA through promoting m6A methylation modification in podocytopathies. Collectively, these findings reveal that METTL14-mediated RNA m6A methylation aggravates proteinuric kidney diseases through targeting Sirt1 mRNA.

## Materials and methods

### Animal models

To establish mice model with ADR nephropathy, adult male C57BL/6J mice (8–12 weeks of age) were purchased from Animal Center of Fudan University and injected with 19.5 mg/kg ADR (D1515, Sigma-Aldrich, St-Louis, MO, USA) intravenously via tail vein. Mice were kept under temperature- and humidity-controlled cages and fed with standard rodent food and water. Proteinuria was measured every week, and mice were sacrificed 6 weeks after injection.

Male type 2 diabetic db/db mice and nondiabetic db/+ mice were commercially obtained from the Jackson Laboratory (Bar Harbor, USA) and fed with sufficient food and water throughout the duration of the experiment. Glycemia and proteinuria were monitored per week and mice were killed at 32 weeks of age.

All mice were randomly allocated to experimental groups. The animal experiments were performed in accordance with the rules of the National Institutes of Health Guide for the Care and Use of Laboratory Animals and were approved by the Institutional Animal Care and Use Committee of Fudan University. All the experiments were replicated at least twice.

### Generation of podocyte-specific METTL14 knockout mice

Homozygous floxed METTL14 (METTL14^fl/fl^) mice on a C57BL/6 background were constructed by Shanghai Model Organisms (Shanghai, China). The Loxp sites were inserted into both sides of Exon3 in METTL14 gene using homologous recombination through CRISPR-Cas9 technology. Nphs2-Cre transgenic mice were provided by the Jackson Research Laboratory (Bar Harbor, USA). Then the METTL14^fl/fl^ mice were crossed with Nphs2-Cre mice to generate podocyte-specific METTL14 deletion mice (METTL14^fl/fl^/Cre^+^). Homozygous floxed METTL14 mice without Cre expression (METTL14^fl/fl^/Cre^−^) were used as controls. Tail genotyping using PCR was performed at the age of 2 weeks. The relevant PCR primer sequences were listed as follows:

LoxP-Forward: CACCTCTGCCTGAACCTCTT;

LoxP-Reverse: TACTAGCTAAACTGATACAACTGG;

Cre-Forward: CGGTTATTCAACTTGCACCA;

Cre-Reverse: GCGCTGCTGCTCCAG.

To investigate the effect of podocyte-specific METTL14 deletion in ADR-treated mice, METTL14^fl/fl^/Cre^+^ and METTL14^fl/fl^/Cre^−^ mice were injected with a single dose of ADR at 19.5 mg/kg via tail vein. Blood, urine and kidney samples were collected 6 weeks after injection.

### Human renal biopsy samples

Our studied human kidney samples included eight cases of DN, six FSGS, six minimal change disease (MCD) and four normal control tissues. The samples of renal biopsies diagnosed with DN, FSGS or MCD were obtained from the Department of Nephrology, Zhongshan Hospital, Fudan University. The control samples were from healthy portions of nephrectomy specimens which were removed for solitary renal cell carcinoma, and they were further validated by histologic examinations and biochemical performance (urine albumin and creatinine ratio < 30 mg/g). The investigator was blind to the group allocation when immunohistochemistry staining for METTL14 (HPA038002, Sigma), WT1 (sc-7385, Santa Cruz Biotechnology, CA, USA) and Sirt1 (ab110304, Abcam, Cambridge, MA, USA) were administrated on these renal tissues. The investigations were conducted under protocols approved by the Clinical Research Ethics Committee of Zhongshan Hospital, Fudan University after the patients provided their informed consent.

### Cell culture and treatments

Conditionally immortalized human podocytes were kindly provided by Dr. John Cijiang He (Icahn School of Medicine at Mount Sinai, New York, USA) and recently authenticated and tested for mycoplasma contamination. Podocytes were cultured in RPMI 1640 (Gibco, MA, USA) supplemented with 10% fetal bovine serum (FBS, Cellsera, NSW, Australia) and 1× insulintransferrin-selenium (ITS, I3146, Sigma-Aldrich) at 33 °C for proliferation. To induce differentiation, cells were maintained at 37 °C for 10 days without ITS. To establish models of podocyte injury in vitro, the differentiated podocytes were starved in medium containing 1% FBS for 12 h and then subjected to different stimuli for 24 h: (1) ADR (0.2, 0.4 or 0.8 μg/ml, D1515, Sigma-Aldrich); (2) high glucose (HG, 25 or 35 mM); (3) AGE (25, 50 or 100 μg/ml). For interference treatment, human podocytes were transfected with negative small interfering RNA (SiNC) or small interfering RNA targeting METTL14 (SiMETTL14) using lipofectamine 3000 (lipo3000, Invitrogen, Carlsbad, CA, USA) following the manufacturer’s instructions. The primer sequence of SiMETTL14 was listed as follows: AGCATTGGTGCCGTGTTAAAT. All the experiments were replicated at least twice.

### Glomerular isolation from mice

Mouse glomeruli were extracted as described [25]. Briefly, mice were perfused with Hank’s balanced salt solution (Gibco, MA, USA) containing 2.5 mg/ml iron oxide and 1% bovine serum albumin (BSA) through the left ventricle. The perfused kidneys were removed, cut into 1 mm^3^ pieces and digested in Hank’s balanced salt solution containing 1 mg/ml collagenase A (Sigma-Aldrich, MO, USA) and 100 U/ml deoxyribonuclease I (Yeasen, Shanghai, China) at 37 °C for 30 min. Next, digested samples were passed through a 100-mm cell strainer and centrifugated for 5 min at 200 × *g*. The pellet was resuspended in HBSS, and glomeruli were extracted using a magnet. The purity of glomeruli was validated by microscopy.

### Global m6A measurement

The m6A levels in total RNAs were colorimetrically quantified by the m6A RNA methylation quantification kit (P-9005-96, Epigentek, Farmingdale, NY, USA). Two hundred nanograms RNAs were coated on per assay well, and the m6A content was captured and detected according to the manufacturer’s instructions. Calculations were conducted based on the standard curve.

### m6A-RNA immunoprecipitation and MeRIP-qPCR

The m6A-RNA immunoprecipitation was performed as previously reported [26, 27]. Briefly, the harvested cells or tissues were collected and lysed with polysome lysis buffer. After the m6A antibody (202003, Synaptic Systems, DE) was bound to coated magnetic beads (Thermo Fisher Scientific, MA, USA), the lysate was incubated with antibody-bead on a rotating wheel overnight at 4 °C. Ten microliters supernatant of the lysate was placed in a new tube labeled “Input” and stored at -80 °C. Next, after digested with proteinase K buffer, the RNAs bound with antibody-bead were extracted by the mixture of phenol, chloroform and isoamyl alcohol. To analyze the enrichment of m6A-modified RNA, quantitative real-time RT-PCR was performed and the m6A-RIP fraction normalized to the input was calculated.

### Dual-luciferase reporter assay

PmirGlo luciferase reporter vector containing firefly luciferase (F-luc) and Renilla luciferase (R-luc) was purchased from Promega (Wisconsin, USA) and used to construct the reporter plasmid. The full-length Sirt1 transcript was inserted into pmirGlo vector after the F-luc coding sequence to construct wild-type Sirt1 reporter plasmid. Transcript with adenine to cytosine mutation within the m6A consensus sequences of Sirt1 was made to construct the mutant Sirt1 reporter plasmid. Cells were seeded in 24-well plates and transfected with wild-type or mutated F-luc-Sirt1 fusion reporter plasmid along with SiNC or SiMETTL14 using lipo3000 reagent. Twenty-four hours after transfection, cells were exposed to different stimuli for another 24 h. Luciferase activity was then evaluated with Dual-Luciferase Reporter Assay System (E1910, Promega, WI, USA) following the manufacturer’s instructions. Expression of Firefly luciferase was used to measure the effect of m6A modification on SIRT1 expression. Renilla Luciferase activity was used as an internal control to normalize the transfection efficiency of the reporter plasmid.

### Assessment of Sirt1 mRNA stability

Podocytes in different groups were treated with 5 μg/ml actinomycin (S8964, Selleck Chemicals, Shanghai, China) for 0, 2, 4 and 6 h. RNA extraction and qRT-PCR was constructed as described earlier [28].

### Assessment of urine albumin and creatinine

Urine albumin was examined by an ELISA kit from Bethyl Laboratories (Texas, USA) following the manufacturer’s protocol. Urine creatinine was evaluated with a QuantiChrom^TM^ Creatinine Assay Kit (Bioassay System, CA, USA) as per the manufacturer’s instructions. The urine albumin excretion rate was determined with the ratio of albumin to creatinine.

### Kidney histology and immunohistochemical staining

The removed kidneys were fixed with 4% paraformaldehyde, embedded in paraffin and then cut into 4-μm-thick sections. Periodic acid–Schiff (PAS, Sigma-Aldrich, USA) staining was performed on the sections for histologic analysis. Glomerulosclerosis scoring was assessed blindly on a minimum of 10 glomeruli per section using a light microscopy (Leica DM 6000B; Leica Microsystems, Wetzeler, Germany).

Immunohistochemical staining for METTL14 (HPA038002, Sigma), WT1 (sc-7385, Santa Cruz Biotechnology) and Sirt1(ab110304, Abcam) was performed. Briefly, after dewaxing, subjected to antigen retrieval and blocked, the sections were incubated with the primary antibodies overnight at 4 °C. The next day, positive staining was revealed by incubating with HRP-conjugated secondary antibodies, diaminobenzidine staining and counterstaining with hematoxylin. Slides were visualized by a light microscopy (Leica DM 6000B; Leica Microsystems, Wetzeler, Germany).

### Immunofluorescence staining

Differentiated human podocytes or frozen kidney sections fixed with 4% paraformaldehyde were blocked and incubated with primary antibodies of WT1 (ab89901, Abcam), synaptopodin (sc-515842, Santa Cruz Biotechnology), METTL14 (HPA038002, Sigma) or Sirt1 (ab110304, Abcam) overnight at 4 °C. Fluorophore-linked secondary antibody (Alexa Fluor 488 anti-mouse IgG or Alexa Fluor 561 anti-rabbit IgG, Invitrogen, Carlsbad, CA) was used to inspect antigen−antibody complexes. Nuclei were counterstained with 4,6-diamidino-2-phenylindole (DAPI) for 15 min. Images were obtained by a confocal microscope (Zeiss LSM 700, Germany).

### Transmission electron microscopy (TEM)

For transmission electron microscopy, 1 mm^3^ kidney cortex samples were fixed in 2.5% glutaraldehyde, postfixed with 1% osmic acid and cut into ultrathin sections. Then the sections were stained with uranyl acetate and lead citrate and photographed by a Hitachi H7650 microscope (Tokyo, Japan). The quantification of foot process (FP) width and glomerular basement membrane (GBM) thickness was performed using ImageJ software (National Institutes of Health, Bethesda, MD) as previously reported. To monitor the autophagy status, the number of typical autophagosomes with double membranes was evaluated in podocytes from mice with different treatment.

### Apoptosis assays

#### TUNEL assay

Apoptosis of podocytes in kidney sections was detected by double immunofluorescence (IF) staining with WT1 (ab89901, Abcam) and TUNEL (Roche, Basel, Switzerland) following the manufacturer’s instructions. Cells with TUNEL and WT1-positive expression were counted on six random fields of each slide using a confocal microscope (Zeiss LSM 700, Germany).

### Flow cytometry

To evaluate podocyte apoptosis in vitro, Annexin V-FITC/PI (BD Bioscience, NJ, USA) double staining on podocytes was conducted as the manufacturer’s protocol. The apoptosis of podocytes with different treatment was detected by flow cytometry (Thermo Fisher Scientific, Pittsburgh, PA, USA) and analyzed by FlowJo 7.6 software.

### Real-time PCR

Total RNA was isolated from harvested cells or renal tissues using Trizol reagent (Invitrogen, Carlsbad, USA). Reverse-transcription to complementary DNA and the followed real-time PCR were performed as previously described [28]. The PCR primer sequences were listed in supplementary materials (Supplementary Table 1). Relative gene expression was analyzed by the 2^−ΔΔCt^ method.

### Western blot

Protein from podocytes and renal tissues was extracted with RIPA buffer containing protease inhibitor cocktail. Equal amounts of protein were separated by SDS-PAGE and transferred to PVDF membranes. Primary antibodies used in this study were listed as follows: anti-METTL3 (ab195352, Abcam), anti-METTL14 (HPA038002, Sigma), anti-WTAP (56501, Cell Signaling Technology, Beverly, MA, USA), anti-FTO (ab92821, Abcam), anti-ALKBH5 (ab195377, Abcam), anti-P62 (39749, Cell Signaling Technology), anti-LC3 (4108S, Cell Signaling Technology), anti-Bcl2 (3498S, Cell Signaling Technology), anti-cleaved caspase3 (9664T, Cell Signaling Technology), anti-caspase3 (9662, Cell Signaling Technology), anti-Sirt1 (ab110304, Abcam), anti-actin (3700, Cell Signaling Technology). Densitometry analysis was performed using ImageJ software (National Institutes of Health, Bethesda, MD).

### Statistical analysis

All experiments were performed in biological replicates. The sample size was estimated based on the need for statistical power. Data were expressed as means ± SEM and statistical analyses were performed using SPSS 21.0 (SPSS Inc., Chicago, IL, USA). Two-tailed, unpaired *t* test was used to analyze the differences between two groups. For multiple comparisons, one-way ANOVA followed by Bonferroni correction was applied. The variance between the groups that are being statistically compared is similar. *P* values < 0.05 were determined to be significant.

## Results

### The increased METTL14-mediated RNA m6A modification in ADR and db/db mice

**Fig. 1:**
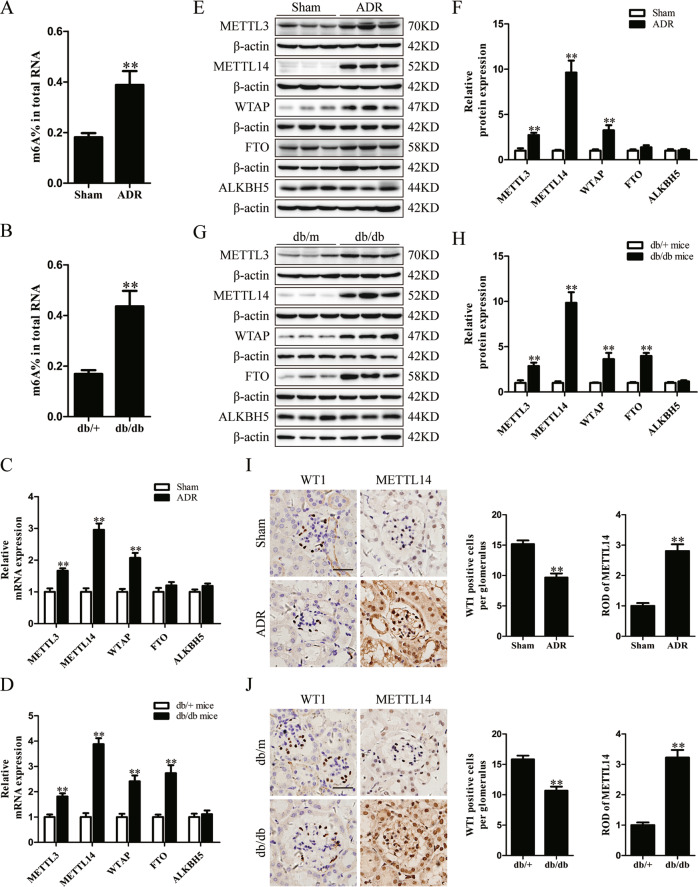
METTL14-mediated RNA m6A modification were upregulated in the renal cortex of mice with ADR and diabetic nephropathy. **A** The m6A levels of total RNAs in the renal cortex from ADR-treated mice and their corresponding controls quantified by colorimetric ELISA assay (*n* = 6). **B** The m6A contents of total RNAs in the renal cortex of diabetic db/db mice and control db/m mice (*n* = 6). **C** The relative mRNA levels of major methyltransferases and demethyltransferases in the renal cortex of mice with ADR treatment or normal controls (*n* = 6). **D** The relative mRNA levels of major m6A modifying enzymes in the renal cortex of db/db mice compared with control db/m mice (*n* = 6). **E**, **F** Western blot analysis of major methyltransferases and demethyltransferases in the renal cortex of saline- or ADR-treated mice (*n* = 6). **G**, **H** Western blot analysis of major m6A modifying enzymes in the renal cortex of db/db mice and control db/m mice (*n* = 6). **I** Representative images and quantification of immunohistochemical staining for METTL14 and WT1 in kidney sections of mice with ADR treatment or normal controls. WT1 were used as podocyte marker. Scale bars, 20 μm (*n* = 6). **J** Images and quantification of immunohistochemical staining for METTL14 and WT1 in kidneys from db/m and db/db mice. Scale bars, 20 μm (*n* = 5). Data are presented as mean ± SEM. **P < 0.01 vs. control group.

To investigate the alteration of RNA m6A modification in proteinuric kidney disease, we quantified the levels of m6A methylated RNA extracted from the renal cortex of ADR-treated mice, diabetic db/db mice and their corresponding controls using colorimetric ELISA assay. As shown in Fig. 1A, B, we found the m6A levels of total RNA were increased in the renal cortex of ADR-treated mice and db/db mice. Next we extracted the glomeruli of mice with the two proteinuric kidney disease, and found the m6A levels of total RNA were also increased in isolated glomeruli of ADR-treated mice and db/db mice compared with their normal controls (Supplementary Fig. 1A, B).

To explore the critical m6A modifying enzymes engaged in the elevated m6A RNA levels, we measured the expression patterns of major methyltransferases (METTL3, METTL14 and WTAP) and demethyltransferases (FTO and ALKBH5) in the renal cortex of ADR-treated mice and db/db mice using qRT-PCR and western blotting. Our results demonstrated that the mRNA and protein levels of METTL3, METTL14 and WTAP were upregulated in the renal cortex of mice receiving ADR treatment. The expression of FTO and ALKBH5 had no significant changes (Fig. 1C, E, F). In contrast, we found compared with db/m mice, the mRNA and protein expression of METTL3, METTL14, WTAP and FTO were elevated in the renal cortex of db/db mice, whereas no obvious differences were observed in the expression of ALKBH5 between the two groups (Fig. 1D, G, H). Interestingly, we identified that the elevation of METTL14 expression was the most evident in the two proteinuric kidney disease models. Additionally, the mRNA levels of major m6A modifying enzymes in isolated glomeruli showed the similar tendency with the aforementioned qRT-PCR results of the renal cortex in ADR-treated mice and db/db mice (Supplementary Fig. 1C, D). Western blot analysis further supported the evident upregulation of METTL14 protein levels in isolated glomeruli of mice with the two proteinuric kidney disease (Supplementary Fig. 1E, F). Our immunohistochemical studies revealed that METTL14 was primarily present in the nuclei of renal cells and dramatically upregulated in kidney sections of ADR-treated mice and db/db mice with the reduction of podocyte marker WT1 expression (Fig. 1I, J). Together, these results indicate METTL14 as the key regulator of the increased m6A RNA modification in the ADR-treated mice and diabetic db/db mice.

### Upregulation of METTL14 in patients with podocytopathies and injured podocytes in vitro

**Fig. 2:**
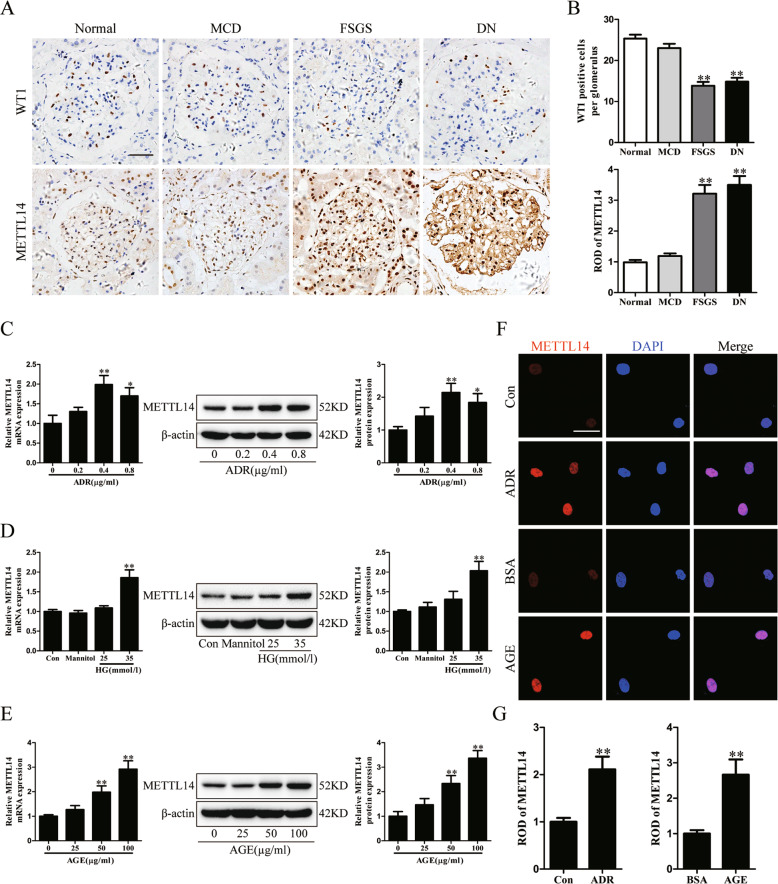
The elevated METTL14 expression in patients with podocytopathies and injured podocytes in vitro. **A**, **B** Representative images and quantification of immunohistochemical staining for METTL14 and WT1 on renal biopsy samples from normal subjects (*n* = 4) and patients with MCD (*n* = 6), FSGS (*n* = 6) and DN (*n* = 8). Scale bars, 20 μm. **P < 0.01 vs. normal subjects. **C** Quantitative RT-PCR and western blot analysis of METTL14 mRNA and protein expression in human cultured podocytes administrated with different doses of ADR (0.2−0.8 μg/ml) for 24 h (*n* = 6). **D** Quantitative RT-PCR and western blot analysis of METTL14 mRNA and protein levels in podocytes subjected to different doses of HG (25 or 35 mmol/l) for 24 h (*n* = 6). **E** Quantitative RT-PCR and western blot analysis of METTL14 mRNA and protein expression in podocytes treated with different doses of AGE (25−100 μg/ml) for 24 h (*n* = 6). **F** Representative confocal microscopic images of immunofluorescence staining for METTL14 in podocytes treated with ADR (0.4 μg/ml) or AGE (50 μg/ml) compared with normal controls. **G** Quantitative analysis of immunofluorescence staining for METTL14 in podocytes subjected to ADR (0.4 μg/ml) or AGE (50 μg/ml) in comparison to normal controls (*n* = 6). Data are presented as mean ± SEM. *P < 0.05, **P < 0.01 vs. control group.

To elucidate the expression of METTL14 in renal tissues of patients with podocytopathies, we performed immunohistochemical staining for METTL14 and WT1 on renal biopsy samples from healthy subjects and patients with MCD, FSGS and DN. Consistent with the aforementioned animal studies, the results revealed that, with the reduction of WT1-positive cells, nuclear METTL14 expression was significantly elevated in biopsy samples of patients with FSGS and DN in comparison to healthy controls or patients with MCD (Fig. 2A, B). Next, we measured the expression of METTL14 in human cultured podocytes subjected to different concentration of ADR, HG or AGE. As shown in Fig. 2C, a suitable concentration of ADR treatment (0.4 μg/ml) induced the upregulation of METTL14 mRNA and protein levels in human cultured podocytes. Similarly, HG or AGE promoted the mRNA and protein expression of METTL14 in a dose-dependent manner in vitro (Fig. 2D, E). Therefore, human podocytes treated with 0.4 μg/ml ADR or 50 μg/ml AGE were selected to simulate ADR or DN respectively in the following studies. Immunofluorescence staining identified METTL14 was mainly presented in nuclei of podocytes and evidently upregulated under ADR or AGE condition (Fig. 2F, G). Moreover, we measured the levels of m6A methylated RNA extracted from cultured human podocytes with ADR or AGE treatment using colorimetric ELISA assay. Consistent with the findings in tissues, the m6A levels of total RNA were elevated in podocytes under ADR or AGE condition compared with their normal controls (Supplementary Fig. 2A, B). Collectively, these data further validate METTL14 as a common pathogenic factor in podocyte injury.

### Podocyte-specific METTL14 deletion improved glomerular function in mice with ADR nephropathy

**Fig. 3:**
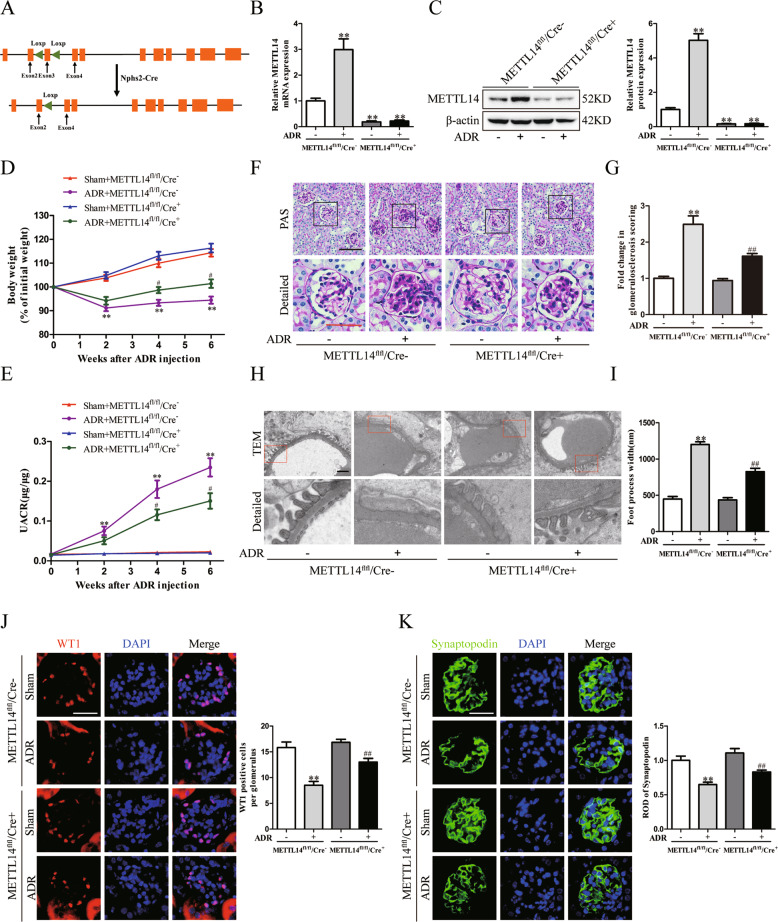
Podocyte-selective deletion of METTL14 alleviated ADR-induced glomerular injury in vivo. Wild-type (METTL14^fl/fl^/Cre^−^) mice and podocyte-specific METTL14 knockout (METTL14^fl/fl^/Cre^+^) mice were injected with saline or ADR (19.5 mg/kg). **A** The diagram depicting generation of METTL14^fl/fl^/Cre^+^ mice. Exon 3 of METTL14 gene were deleted in podocytes using Cre–LoxP recombination system. **B** The relative mRNA levels of METTL14 in isolated glomeruli from different groups of mice (*n* = 4). **C** Representative immunoblots and quantitative analysis showing the evident decreased protein levels of METTL14 in isolated glomeruli from METTL14^fl/fl^/Cre^+^ mice with ADR injection (*n* = 4). **D** Quantitative analysis of body weight in different groups of mice (*n* = 6). **E** UACR (urine albumin-to creatinine ratio) in each group (*n* = 6). **F** Representative images of PAS staining on kidney sections in different groups of mice. Scale bar: black 40 μm, red 20 μm. **G** Assessment of morphologic injury quantified with messangial matrix expansion in each group (*n* = 6). **H** Representative transmission electron microscopy images of podocyte foot process in different groups of mice. Scale bar, 1 μm. **I** Quantitative analysis of podocyte foot process effacement and GBM thickness in the four groups (*n* = 6). **J** Representative images and quantification of immunofluorescence staining for WT1 on kidneys of each group. Scale bar, 20 μm (*n* = 6). **K** Representative images and quantification of immunofluorescence staining for synaptopodin on kidneys of each group. Scale bar, 20 μm (*n* = 6). **P < 0.01 vs. METTL14^fl/fl^/Cre^−^ mice injected with saline, ^#^*P* < 0.05, ^##^*P* < 0.01 vs. METTL14^fl/fl^/Cre^−^ mice with ADR injection. Data are presented as mean ± SEM.

To delineate the role of METTL14 in maintaining podocyte function, we generated a conditional mouse model where METTL14 was specifically deleted in podocytes using the Cre-Loxp system. As shown in Fig. 3A, Podocin-Cre mice were bred with METTL14-floxed mice to generate podocyte-specific METTL14 ablated mice (METTL14^flox/flox^/Cre^+^). METTL14^flox/flox^/Cre^−^ mice were used as wild-type mice. We then conducted single ADR injection (19.5 mg/kg) to the METTL14^flox/flox^/Cre^+^ and METTL14^flox/flox^/Cre^−^ mice respectively. Deletion of METTL14 in podocytes was verified by evaluating METTL14 mRNA and protein levels in isolated glomeruli from mice with different treatment using qRT-PCR and western blotting. Compared with ADR-treated METTL14^flox/flox^/Cre^−^ mice, the mRNA and protein expression of METTL14 was evidently inhibited in the glomeruli isolated from ADR-treated METTL14^flox/flox^/Cre^+^ mice (Fig. 3B, C).

Next we measured glomerular function of podocyte-specific METTL14 knockout mouse model with ADR nephropathy. As shown in Fig. 3D, METTL14^flox/flox^/Cre^+^ mice displayed ameliorated weight loss in extent to that of METTL14^flox/flox^/Cre^−^ mice after ADR treatment. We then analyzed urinary albumin excretion and observed a marked reduction of urinary albumin-to-creatinine ratio (UACR) in ADR-treated METTL14^flox/flox^/Cre^+^ mice as compared with ADR-treated METTL14^flox/flox^/Cre^−^ mice (Fig. 3E). PAS staining demonstrated a slighter degree of glomerulosclerosis in kidney sections from METTL14^flox/flox^/Cre^+^ mice with ADR treatment compared with ADR-treated METTL14^flox/flox^/Cre^−^ mice (Fig. 3F, G). TEM analysis revealed ADR intervention resulted in exacerbated podocyte foot process broadening and effacement in METTL14^flox/flox^/Cre^−^ mice. The deteriorated ultrastructural changes of podocytes induced by ADR injection were significantly improved in mice with podocyte-specific METTL14 deletion (Fig. 3H, I). The severity of podocyte injury was further observed by immunostaining of podocyte-specific markers including WT1 and synaptopodin. In comparison to ADR-treated METTL14^flox/flox^/Cre^−^ mice, the number of WT1-positive cells and the expression of synaptopodin were significantly upregulated in kidney sections of METTL14^flox/flox^/Cre^+^ mice with ADR injection (Fig. 3J, K). With the above evidence, we conclude that podocyte-selective deletion of METTL14 ameliorates glomerular injury in mice with nephropathy.

### METTL14 deletion in podocytes facilitated autophagy and ameliorated apoptosis and inflammation in ADR mice

**Fig. 4:**
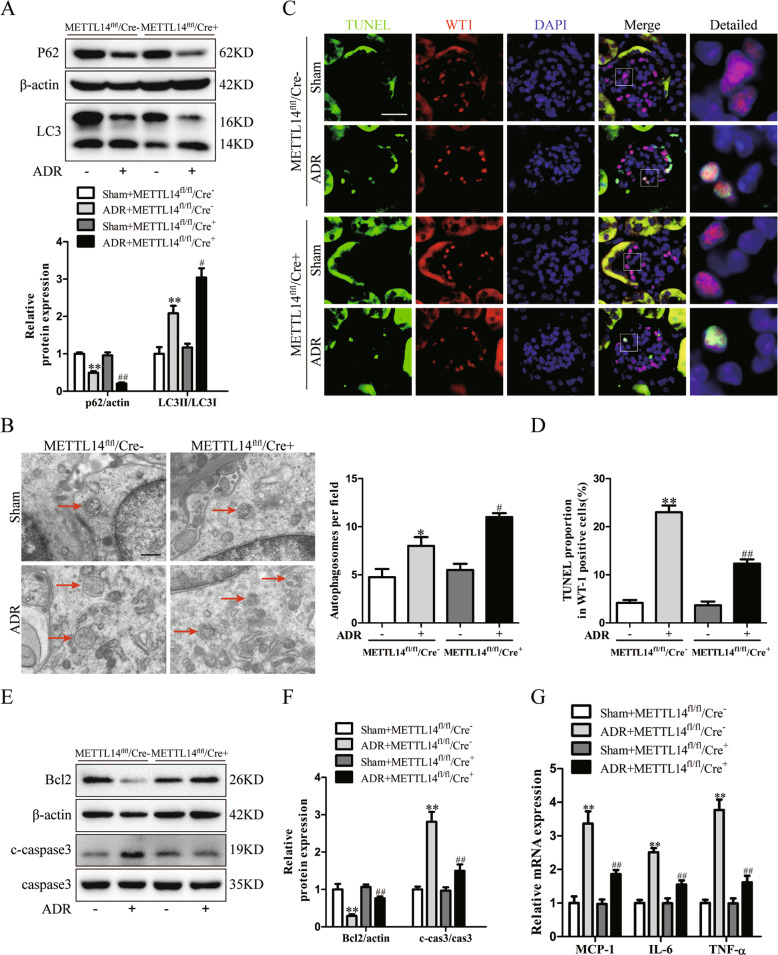
Podocyte-specific METTL14 deletion promoted autophagy and inhibited apoptosis and inflammation in mice with ADR nephropathy. Wild-type (METTL14^fl/fl^/Cre^−^) mice and podocyte-specific METTL14 knockout (METTL14^fl/fl^/Cre^+^) mice were injected with saline or ADR (19.5 mg/kg). **A** Representative immunoblots and quantitative analysis of autophagy-related proteins (P62 and LC3 II/LC3 I) in the renal cortex from different groups of mice (*n* = 6). **B** Representative electronic microscopy images and statistical analysis of typical autophagosomes (red arrow) in each group. Scale bar, 500 nm (*n* = 4). **C** Representative immunofluorescence images of TUNEL and WT1 double staining on kidney sections from different groups of mice. Nuclei were stained with DAPI (blue). Green fluorescence indicates TUNEL-positive nuclei. Red fluorescence represents WT1-positive nuclei. Scale bar, 20 μm. **D** Statistical analysis of apoptotic podocytes with TUNEL-positive and WT1-positive staining in kidney sections from each group (*n* = 6). **E** Representative immunoblots of proteins associated with apoptosis in the renal cortex of each group. **F** Quantitative analysis of Bcl2 and cleaved caspase-3 in these groups (*n* = 6). **G** Quantitative RT-PCR analysis of inflammatory cytokines mRNA levels (MCP-1, IL-6 and TNF-α) in groups of mice with different treatment (*n* = 6). *P < 0.05, **P < 0.01 vs. METTL14^fl/fl^/Cre^−^ mice injected with saline, ^#^*P* < 0.05, ^##^*P* < 0.01 vs^.^ METTL14^fl/fl^/Cre^−^ mice with ADR injection. Data are presented as mean ± SEM.

To determine the effect of podocyte-specific METTL14 knockout on autophagy status in mice with ADR nephropathy, we examined autophagy-related proteins in the renal cortex of mice with different treatment. Consistent with previous studies [29], western blot analysis demonstrated that ADR injection promoted the degradation of autophagy substrate p62 and upregulated LC3 II/LC3 I expression ratio in wild-type mice. Podocyte-specific METTL14 knockout further improved P62 degradation and LC3-I to LC-3II conversion in the renal cortex of mice with ADR nephropathy (Fig. 4A). Then we used TEM to observe the typical autophagosomes with double membranes in podocytes from mice with different treatment. As shown in Fig. 4B, the number of autophagosomes was elevated in podocytes from METTL14^flox/flox^/Cre^−^ mice after ADR injection, and this number was further elevated in podocytes from ADR-treated METTL14^flox/flox^/Cre^+^ mice.

Next, we evaluated the role of podocyte-specific METTL14 deletion in podocyte apoptosis and renal inflammation induced by ADR injection. Immunofluorescence co-staining of TUNEL and podocyte marker WT1 revealed the evident reduction of podocyte apoptosis in ADR-treated METTL14^flox/flox^/Cre^+^ mice when compared to ADR-treated METTL14^flox/flox^/Cre^−^ mice (Fig. 4C, D). The immublotting results revealed that the expression of antiapoptotic Bcl2 was inhibited and the level of proapoptotic cleaved caspase-3 was upregulated in the renal cortex of ADR-treated METTL14^flox/flox^/Cre^−^ mice. Podocyte-specific METTL14 deletion suppressed the effect of ADR injection on Bcl2 and cleaved caspase-3 expression in the renal cortex of mice (Fig. 4E, F). Moreover, qRT-PCR analysis found METTL14 deletion in podocytes inhibited the upregulation of proinflammatory mediators (MCP-1, IL-6 and TNF-α) mRNA levels in the renal cortex of mice with ADR treatment (Fig. 4G). These data provide evidence that podocyte-specific METTL14 deletion alleviates podocyte injury by promoting autophagy and inhibiting apoptosis and inflammation in mice with ADR nephropathy.

### METTL14 knockdown suppressed ADR-induced podocyte injury in vitro

**Fig. 5:**
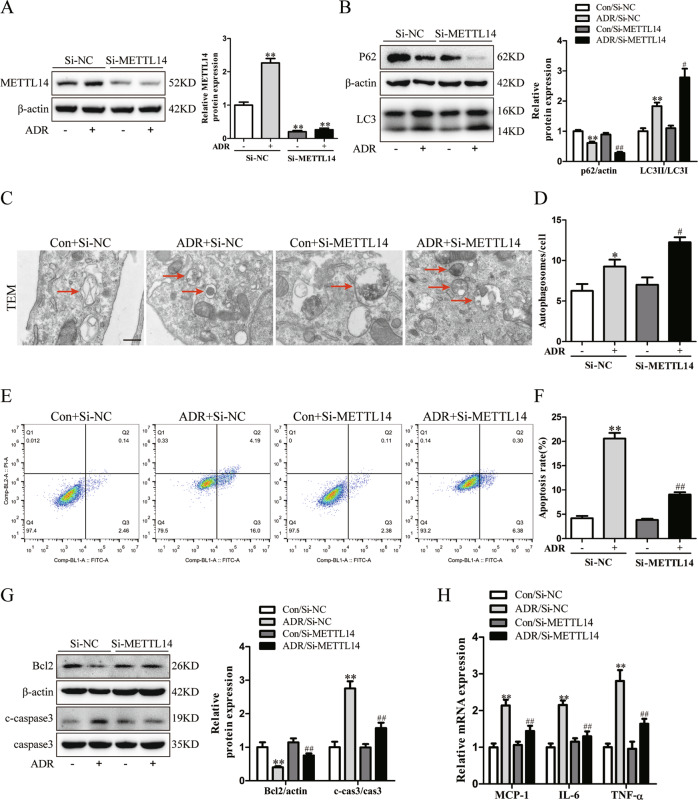
Knockdown of METTL14 attenuated podocyte injury induced by ADR in vitro. Podocytes transfected with negative SiRNA (SiNC) or METTL14 knockdown SiRNA (SiMETTL14) were subjected to saline or ADR (0.4 μg/ml). **A** Western blot analysis of METTL14 protein expression in podocytes with different treatments (*n* = 6). **B** Western blot analysis showing further promotion of autophagy substrate P62 degradation and LC3-I to LC-3II conversion induced by METTL14 knockdown in ADR-treated podocytes (*n* = 6). **C** Representative electronic microscopy images of typical autophagosomes in podocytes with different treatments. The arrows indicate typical autophagosomes with double membranes. Scale bar, 500 nm. **D** Quantitative analysis of autophagosomes/cell in each group (*n* = 4). **E** Cell apoptosis in podocytes with different treatments. Podocytes in each group were stained with FITC-conjugated Annexin V and PI and examined by flow cytometry. Annexin V-positive podocytes were quantified as apoptotic cells. **F** Summarized data showing the apoptosis rate of podocytes analyzed by FlowJo software in each group (*n* = 6). **G** Representative immunoblots and quantitative analysis of proapoptotic cleaved caspase-3 and antiapoptotic Bcl2 in the four groups (*n* = 6). **H** The mRNA levels of proinflammatory mediators (MCP-1, IL-6 and TNF-α) in different groups of podocytes (*n* = 6). *P < 0.05, **P < 0.01 vs. Con + SiNC group, ^#^*P* < 0.05, ^##^*P* < 0.01 vs. ADR + SiNC group. Data are presented as mean ± SEM.

To address the role of METTL14 in podocytes receiving ADR administration in vitro, the cultured human podocytes were transfected with SiNC or SiMETTL14, and then subjected to saline or ADR (0.4 μg/ml). Knockdown efficiency was confirmed by western blot analysis (Fig. 5A). Then we explored the effect of METTL14 on regulating podocyte autophagy under ADR condition in vitro. The immubloting results demonstrated that the promotion of P62 degradation and LC3-I to LC-3II conversion was further augmented in podocytes with METTL14 knockdown under ADR condition (Fig. 5B). Using TEM, we found the increased number of autophagosomes induced by ADR intervention was further upregulated in podocytes with METTL14 knockout (Fig. 5C, D). These findings indicate METTL14 knockdown contributes to the further activation of autophagy in ADR-treated podocytes.

To evaluate the effect of METTL14 in ADR-induced podocyte apoptosis in vitro, we performed FITC-Annexin V and PI staining on podocytes and analyzed the apoptotic rate using flow cytometry. Quantitatively, ADR treatment resulted in an evident increase in the percentage of apoptotic podocytes. Knockdown of METTL14 attenuated the proapoptotic effect of ADR on podocytes (Fig. 5E, F). Additionally, immubloting results revealed that podocytes with METTL14 knockdown displayed increased protein level of antiapoptotic Bcl2 and reduced expression of proapoptotic cleaved caspase-3 in comparison to podocytes transfected with SiNC under ADR condition (Fig. 5G). These results suggest METTL14 suppression protects podocytes from apoptosis induced by ADR intervention. Finally, we detected the mRNA levels of the inflammatory cytokines (MCP-1, IL-6 and TNF-α) using qRT-PCR and found METTL14 knockdown blunted the elevation of proinflammatory mediators in podocytes with ADR treament (Fig. 5H), which indicate the anti-inflammatory effect of METTL14 suppression on ADR-treated podocytes.

### METTL14 silencing has pleiotropic protective actions in podocytes with AGE treatment

**Fig. 6:**
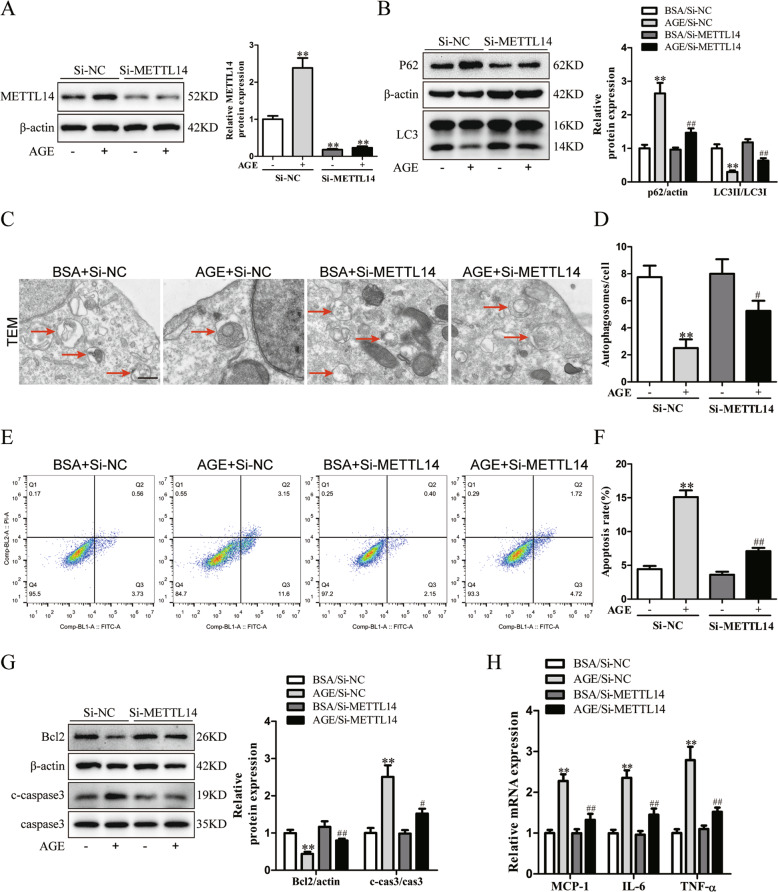
Gene silencing of METTL14 protected against AGE-induced podocyte injury in vitro. Podocytes transfected with negative SiRNA (SiNC) or METTL14 knockdown SiRNA (SiMETTL14) were administrated with BSA or AGE (50 μg/ml). **A** Representative immunoblots and quantitative analysis of METTL14 in different groups of podocytes (*n* = 6). **B** Western blot analysis showing METTL14 knockdown weakened AGE-induced inhibition of P62 degradation and LC3-I to LC-3II conversion in podocytes (*n* = 6). **C**, **D** Representative electronic micrographs and quantitative analysis showing METTL14 silencing ameliorated the reduction of typical autophagosomes in podocytes induced by AGE treatment. Scale bar, 500 nm (*n* = 4). **E**, **F** Apoptosis of podocytes with different treatments analyzed by flow cytometry. The results showed that METTL14 knockdown downregulated the percentage of apoptotic podocytes in AGE-treated podocytes (*n* = 6). **G** Western blot analysis of apoptosis-related proteins (Bcl2 and cleaved caspase3) in podocytes with different treatment (*n* = 6). **H** Quantitative RT-PCR analysis showing the reduction of inflammatory cytokines (MCP-1, IL-6 and TNF-α) mRNA levels with METTL14 inhibition in AGE-treated podocytes (*n* = 6). **P < 0.01 vs. BSA + SiNC group, ^#^*P* < 0.05, ^##^*P* < 0.01 vs. AGE + SiNC group. Data are presented as mean ± SEM.

The cultured human podocytes transfected with SiNC or SiMETTL14 were administrated with BSA or AGE (50 μg/ml). Then we validated the knockdown efficiency of METTL14 in podocytes with different treatment by western blotting (Fig. 6A). Previous studies have reported the decreased autophagy level of podocytes is closely associated with podocyte dysfunction in DN [30]. As shown in Fig. 6B, AGE treatment inhibited P62 degradation and suppressed LC3-I to LC-3II conversion in podocytes. METTL14 gene silencing restored the alteration of autophagy-related proteins in podocytes with AGE intervention. Moreover, we found METTL14 knockdown suppressed the reduction of autophagosomes in podocytes receiving AGE treatment through TEM (Fig. 6C, D). These findings suggest METTL14 silencing can restore podocyte autophagy inhibited by AGE intervention.

Next we quantified the apoptosis of podocytes using FITC-Annexin V and PI staining. Flow cytometry analysis revealed that METTL14 silencing inhibited the upregulation of apoptotic podocytes after AGE treatment (Fig. 6E, F). In addition, western blot analysis demonstrated that AGE intervention elevated the level of proapoptotic cleaved caspase-3 and reduced expression of antiapoptotic Bcl2, which was attenuated by METTL14 silencing in podocytes (Fig. 6G). These results reveal the antiapoptotic effect of METTL14 knockdown under AGE condition. To explore the role of METTL14 on inflammatory response in AGE-treated podocytes, the mRNA levels of proinflammatory mediators were evaluated in different groups. The results demonstrated that METTL14 knockdown reduced the effect of AGE on elevating mRNA levels of MCP-1, IL-6 and TNF-α (Fig. 6H), indicating the protective role of METTL14 inhibition in inflammatory response of podocytes under AGE condition.

### METTL14 deletion inhibited Sirt1 mRNA m6A modification and degradation in injured podocytes

**Fig. 7:**
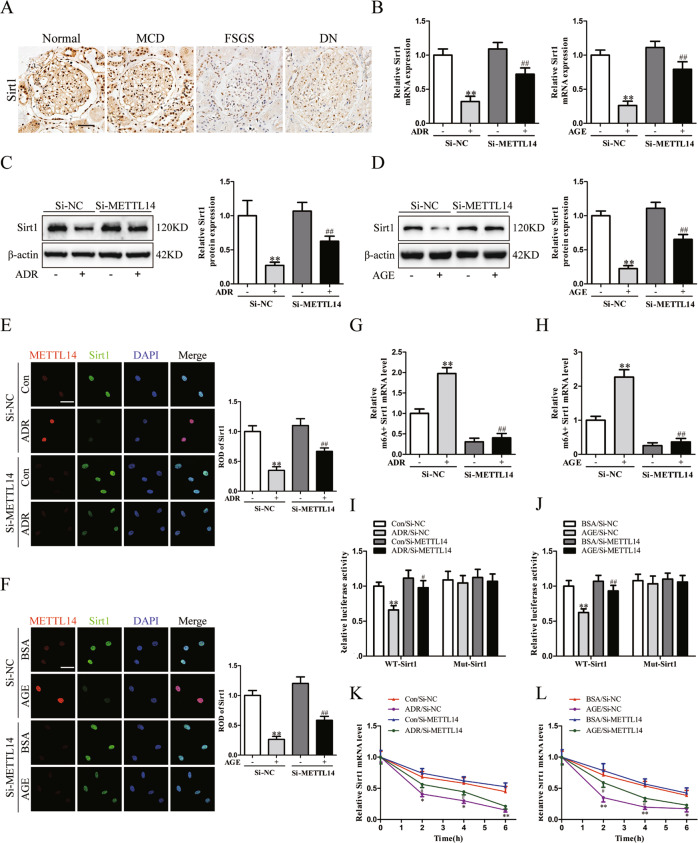
The protective effect of METTL14 knockdown in injured podocyte relied on promoting Sirt1 mRNA m6A modification and degradation. **A** Representative images of immunohistochemical staining for Sirt1 on renal biopsies from normal subjects (*n* = 4) and patients with MCD (*n* = 6), FSGS (*n* = 6) and DN (*n* = 8). Scale bars, 20 μm. **B** The relative mRNA levels of Sirt1 in podocytes transfected with SiNC or SiMETTL14 under ADR (0.4 μg/ml) or AGE (50 μg/ml) condition for 24 h (*n* = 6). **C** Western blot analysis showing the upregulation of Sirt1 expression in ADR-treated podocytes (*n* = 6). **D** Western blot analysis showing METTL14 knockdown weakened AGE-induced inhibition of Sirt1 expression in podocytes (*n* = 6). **E**, **F** Representative confocal microscopic images and quantification of immunofluorescence co-staining for METTL14 and Sirt1 in podocytes transfected with SiNC or SiMETTL14 under ADR or AGE condition. Scale bar, 40 μm (*n* = 6). **G**, **H** MeRIP-qPCR analysis in podocytes with different treatment. The results indicated ADR or AGE intervention promoted m6A antibody binding with Sirt1 mRNA. METTL14 silencing ameliorated the upregulation of methylated Sirt1 mRNA levels induced by ADR or AGE treatment (*n* = 5). **I**, **J** Luciferase assays were performed in podocytes transfected with wild-type or mutant Sirt1 reporter plasmids under different condition. AGE or ADR administration attenuated the luciferase activity of podocytes transfected with wild-type Sirt1 reporter plasmids. METTL14 knockdown in podocytes relieved the reduction of luciferase activity induced by AGE or ADR treatment. However, ADR or AGE intervention had no effect on the luciferase activity of podocytes transfected with mutant Sirt1 reporter plasmids (*n* = 5). **K**, **L** Podocytes with different treatment were followed by incubation with actinomycin D for 0, 2, 4, 6 h. The decay curves of Sirt1 mRNA were shown. All values are normalized as fractions of the original mRNA levels (*n* = 6). *P < 0.05, **P < 0.01 vs. Con/BSA + SiNC group, ^#^*P* < 0.05, ^##^*P* < 0.01 vs^.^ ADR/AGE + SiNC group. Data are presented as mean ± SEM.

It is known that Sirt1 exerts a critical protective role in alleviating proteinuric kidney disease[18]. Consistent with previous studies [21], our immunohistochemistry staining results validated the reduction of Sirt1 expression in renal biopsies from patients with FSGS and DN in comparison to normal subjects or patients with MCD (Fig. 7A). We then quantified the m6A modification of Sirt1 mRNA in isolated glomeruli of wild-type mice and podocyte-specific METTL14 knockout mice using MeRIP-qPCR. The results demonstrated the reduced m6A methylated levels of Sirt1 mRNA in isolated glomeruli from METTL14^flox/flox^/Cre^+^ mice compared with those from wild-type mice (Supplementary Fig. 3). Thus, we speculate Sirt1 is a downstream target of METTL14-mediated RNA m6A modification in podocytopathies.

To validate our inference, we explored the effect of METTL14 on Sirt1 expression in cultured human podocytes treated with ADR or AGE. QRT-PCR and western blotting analysis demonstrated that METTL14 deletion could restore the mRNA and protein levels of Sirt1 which was inhibited by ADR or AGE treatment in vitro (Fig. 7B−D). Immunofluorescence staining for Sirt1 and METTL14 in podocytes with different treatment further validated the inhibited Sirt1 expression induced by ADR or AGE was significantly upregulated with METTL14 silencing in podocytes. (Fig. 7E, F). Moreover, to explore the mechanism of METTL14 downregulating Sirt1 in injured podocytes, we used MeRIP-qPCR and found ADR or AGE treatment promoted m6A-specific antibody binding with Sirt1 mRNA in podocytes compared with normal control. Deletion of METTL14 evidently relieved the upregulation of methylated Sirt1 mRNA level resulted from ADR or AGE intervention on podocytes (Fig. 7G, H). To further confirm the effect of METTL14-mediated m6A modification on Sirt1 mRNA, we preformed dual-luciferase assays on podocytes transfected with wild-type and mutant Sirt1 reporter plasmid. The adenine residue embedded within m6A consensus sequences (i.e., RRACH), which was located around stop codon in Sirt1 CDS, was replaced by cytosine to construct the mutant reporter plasmid. As shown in Fig. 7I, J, ADR or AGE intervention downregulated the luciferase activity in podocytes transfected with wild-type Sirt1 reporter plasmid. METTL14 silencing recovered the reduction of luciferase activity resulted from ADR or AGE administration. Interestingly, ADR or AGE treatment had no effect on the luciferase activity in podocytes transfected with mutant Sirt1 reporter plasmid, indicating the mutation prevented METTL14-mediated m6A modification on Sirt1 mRNA. We next detected whether m6A modification affected Sirt1 mRNA stability using actinomycin D. The results demonstrated that METTL14 knockdown evidently suppressed the elevation of Sirt1 mRNA decay rate in podocytes treated with ADR or AGE, suggesting METTL14-mediated m6A modification downregulates Sirt1 mRNA stability in injured podocytes (Fig. 7K, L). Taken together, these findings reveal METTL14 aggravates progression of podocytopathies through promoting Sirt1 mRNA m6A modification and degradation.

## Discussion

**Fig. 8:**
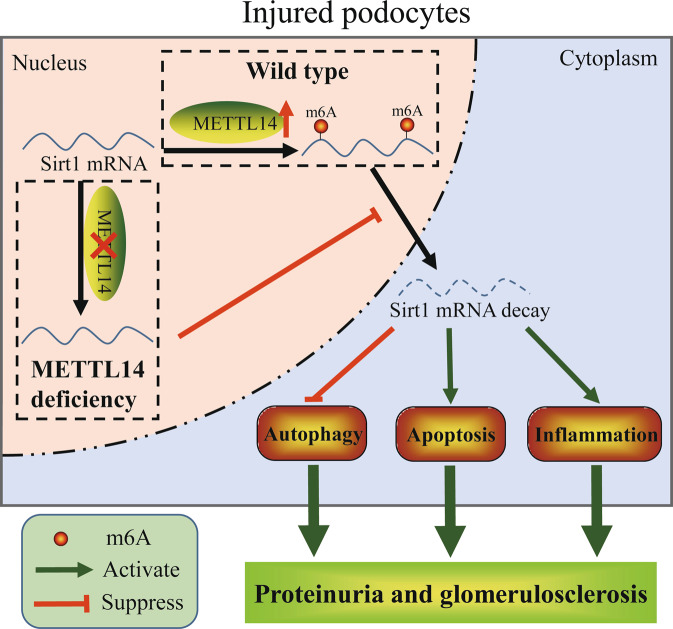
Proposed schematic depicting the mechanism of METTL14 deficiency in alleviating podocyte injury. Podocyte injury upregulates the expression of METTL14, which results in the elevation of Sirt1 mRNA m6A modification and degradation. METTL14 knockdown leads to the inhibition of Sirt1 mRNA m6A modification, thereby increasing Sirt1 levels in injured podocytes. The upregulation of Sirt1 levels finally results in the activation of autophagy and the reduction of apoptosis and inflammation in podocytes, consequently alleviating proteinuria and delaying progression of podocytopathies.

Podocyte injury is known as one of the most important determinants in the progression of proteinuric kidney disease [31, 32]. Investigating the underlying mechanism of podocyte injury is significant for exploring new effective therapeutic strategies on podocytopathies [4]. Although emerging evidence have indicated the significance of m6A methylation on regulating various biological and pathological processes [33, 34], limited studies have elucidated its potential in the regulation of renal function. Here, we first unveiled the upregulation of METTL14-mediated RNA m6A modification in renal tissues of mice with ADR or DN and patients with FSGS and DN. Knockdown of METTL14 had a protective effect on injured podocytes by promoting autophagy and alleviating apoptosis and inflammation both in vivo and in vitro. Moreover, we identified METTL14 aggravated podocyte injury through promoting Sirt1 mRNA m6A modification and degradation (Fig. 8).

In this study, we analyzed the expression patterns of major methyltransferases and demethyltransferases in kidneys of mice with ADR or DN. We observed the elevated expression of METTL3, METTL14 and WTAP in ADR-treated mice. However, the levels of METTL3, METTL14, WTAP and FTO were increased in kidneys of db/db mice in comparison to db/m mice. Considering the rising m6A RNA levels and the most evident upregulation of METTL14 expression in kidneys of the two murine models for proteinuric kidney disease, methyltransferases METTL14 was chosen as the target in our study. The functional studies validated the protective effect of METTL14 knockdown on podocytes both in vivo and in vitro. Our findings are consistent with the previous studies that reveal the pathogenic effect of METTL14 in renal ischemia-reperfusion injury [16] and vascular calcification induced by indoxyl sulfate [17]. However, we also observed the alteration of other major m6A modifying enzymes, such as METTL3 and FTO, in proteinuric kidney disease models as mentioned above. Further investigations would be needed to find out whether these m6A-regulating enzymes contribute to the progression of podocytopathies. In addition, our results provided evidence that the assessment of the m6A RNA levels and METTL14 expression can be used as a novel diagnostic biomarker of podocytopathies with the innovation of detection technology. Further investigation would be needed to elaborate the potential diagnostic value of m6A modification in proteinuric kidney disease.

Except that METTL14 knockdown had anti-apoptosis and anti-inflammation effect on podocytes under pathological condition, we identified METTL14 deletion could modulate autophagy status in podocytes. Podocytes, as highly differentiated and long-lived cells in kidneys, are known to display a high level of basal autophagy for homeostasis maintenance [35, 36]. Early evidence have demonstrated autophagy activation protect mice against podocyte injury and suppress the progression of podocytopathies [37, 38]. Specific ablation of autophagy gene Atg5 result in albuminuria and glomerulopathy in aging mice [39] and podocyte deletion of Atg7 exaggerate ADR-induced podocyte injury [29]. Interestingly, our data indicated that autophagy was activated in ADR-treated podocytes while autophagy inhibition was observed in podocytes under AGE condition, which were consistent with previous studies [29, 30]. We conferred that autophagy activation could be a compensatory protective reaction to cell stress in early stage of ADR-treated podocytes. Functionally, we found METTL14 knockdown promoted podocyte autophagy no matter under AGE or ADR condition, indicating the protective role of METTL14 deletion against podocyte injury. ADR-treated METTL14^fl/fl^-Cre^+^ mice demonstrated an evident increase in the level of autophagy as compared with ADR-treated METTL14^fl/fl^-Cre^−^ mice, further validating METTL14 knockdown positively regulated podocyte autophagy in the pathogenesis of podocytopathies.

Sirt1 is a well-known functional gene involved in renal diseases through regulating various cellular biological process, such as apoptosis, autophagy, inflammation and mitochondrial biogenesis [40, 41]. Although emerging evidence have highlighted the importance of posttranscriptional modification, such as transcription factors and non-coding small RNAs, in regulating Sirt1 downregulation in disease models [42, 43], the effect of m6A modification on Sirt1 mRNA have not been elucidated yet. In our study, we identified that METTL14-mediated m6A modification epigenetically promoted Sirt1 mRNA degradation in podocytopathies. QRT-PCR, western blotting and immunofluorescence staining revealed METTL14 knockdown upregulated Sirt1 expression in podocytes with ADR or AGE treatment. The results of MeRIP-qPCR, luciferase assay and actinomycin D experiments further indicated METTL14 promoted Sirt1 mRNA m6A modification and degradation in injured podocytes. It is noteworthy that m6A modification is recognized by m6A reader proteins to exert its function [5]. Recent studies have reported several m6A reader proteins, such as YT521-B homology (YTH) domain family proteins, heterogeneous nuclear ribonucleoprotein (hnRNP) and eukaryotic initiation factor (eIF) [44, 45]. Among which the human YTH domain family 2 (YTHDF2) protein was reported to mediate mRNA decay by binding to its targets with a conserved core motif of G(m6A)C [46]. The C-terminal domain of YTHDF2 binds to m6A sites and the N-terminal domain facilitates the YTHDF2-mRNA complex transferring to cellular RNA decay sites [47]. Thus, the role of m6A readers, especially YTHDF2, in METTL14-mediated Sirt1 downregulation under pathological conditions, attracts our great interest and still needs further investigation.

Collectively, our work reveal the elevation of METTL14-dependent RNA m6A modification aggravate podocyte injury and proteinuria through posttranscriptional regulating Sirt1 mRNA stability. Our findings indicate dysregulated RNA m6A modification mediated by METTL14 may be a promising target for the diagnosis and therapy of proteinuric kidney disease.

## Supplementary information

Supplemental Figure 1

Supplemental Figure 2-3

Supplemental Figure legend

Supplemental Table

## Data Availability

All data generated in the study are included in this article and its supplementary information files.
