# Supplementary figures and images for: METTL14 aggravates podocyte injury and glomerulopathy progression through N6-methyladenosine-dependent downregulating of Sirt1
Source: Cell Death Dis. 2021 Sep 27;12(10):881. doi: 10.1038/s41419-021-04156-y (PMC8476597; doi:10.1038/s41419-021-04156-y)

A

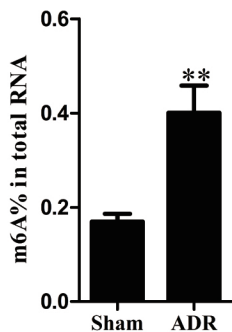

B

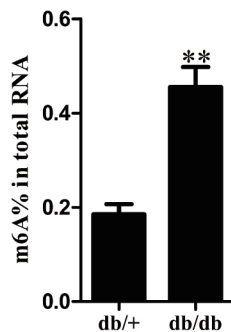

C

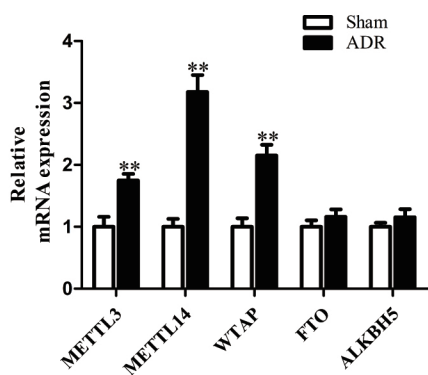

D

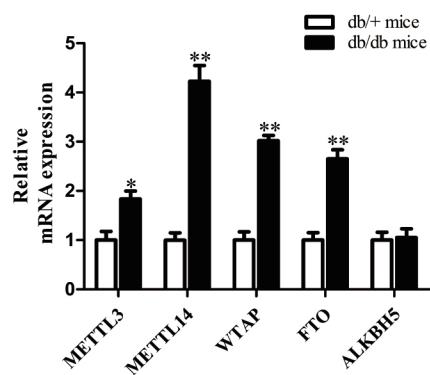

E

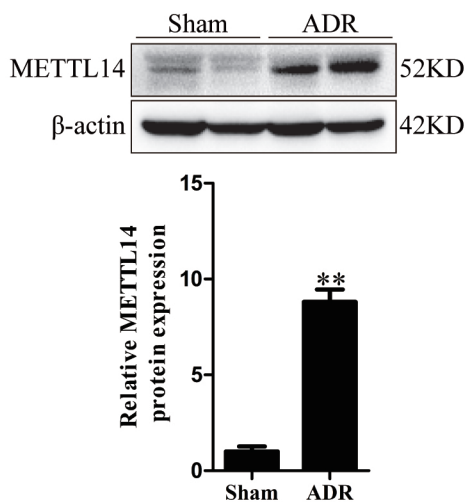

F

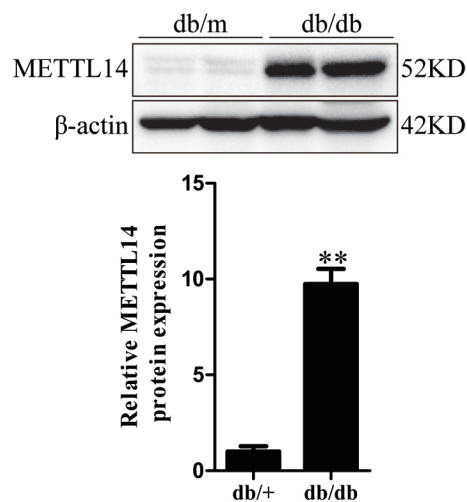

Supplement: Supplementary file 1 — Supplemental Figure 1 [file 41419_2021_4156_MOESM1_ESM.pdf]

Fig. S2A

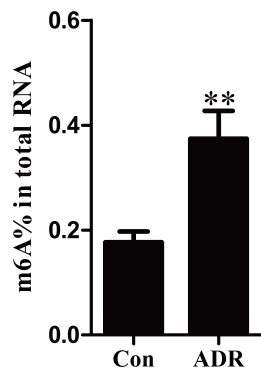

Fig. S2B

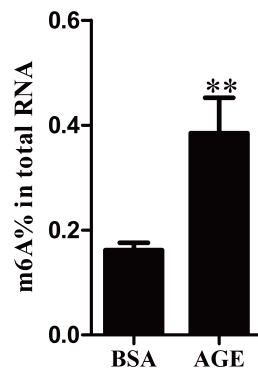

Fig. S3

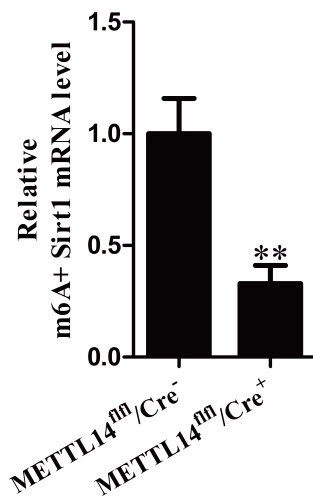

Supplement: Supplementary file 2 — Supplemental Figure 2-3 [file 41419_2021_4156_MOESM2_ESM.pdf]
