# Supplementary material for: METTL14 aggravates podocyte injury and glomerulopathy progression through N6-methyladenosine-dependent downregulating of Sirt1
Source: Cell Death Dis. 2021 Sep 27;12(10):881. doi: 10.1038/s41419-021-04156-y (PMC8476597; doi:10.1038/s41419-021-04156-y)
Supplement: Supplementary file 3 — Supplemental Figure legend [file 41419_2021_4156_MOESM3_ESM.pdf]

Supplemental Figure Legend

Fig. S1. The elevated METTL14-mediated RNA m6A modification in isolated glomeruli from ADR-treated mice and diabetic db/db mice. (A) The m6A levels of total RNAs in isolated glomeruli from kidneys of ADR-treated mice and their corresponding controls quantified by colorimetric ELISA assay. (n=4). (B) The m6A contents of total RNAs in isolated glomeruli from kidneys of diabetic db/db mice and control db/m mice. (n=4). (C) Quantitative RT-PCR analysis of major methyltransferases and demethyltransferases in isolated glomeruli from mice with ADR treatment or normal controls. (n=4.) (D) Quantitative RT-PCR analysis of major m6A modifying enzymes in isolated glomeruli from db/db mice compared with control db/m mice. (n=4). (E) Representative western blots and statistical analysis of METTL14 in isolated glomeruli from kidneys of saline- or ADR-treated mice. (n=4). (F) Representative western blots and statistical analysis of METTL14 in isolated glomeruli from kidneys of db/db mice and control db/m mice. (n=4). Data are presented as mean ± SEM. $^{*}P < 0.05$, $^{**}P < 0.01$ vs. control group.

Fig. S2. The up-regulated RNA m6A levels in human cultured podocytes under ADR or AGE condition. (A) The m6A levels of total RNAs in human cultured podocytes with 0.4 μg/ml ADR treatment and their normal controls quantified by colorimetric ELISA assay. (n=6). (B) The m6A contents of total RNAs in human cultured podocytes treated with 50 μg/ml AGE or BSA. (n=6).

Fig. S3. The decreased m6A modification of Sirt1 mRNA in isolated glomeruli of mice with podocyte-specific METTL14 deletion. Relative methylated Sirt1 mRNA levels in isolated glomeruli from METTL14$^{flox/flox}$/Cre^{+} mice compared with those from wild-type mice. (n=4).
