# Supplementary material for: METTL14 aggravates podocyte injury and glomerulopathy progression through N6-methyladenosine-dependent downregulating of Sirt1
Source: Cell Death Dis. 2021 Sep 27;12(10):881. doi: 10.1038/s41419-021-04156-y (PMC8476597; doi:10.1038/s41419-021-04156-y)
Supplement: Supplementary file 4 — Supplemental Table [file 41419_2021_4156_MOESM4_ESM.pdf]

Supplemental Table. Primers for quantitative RT-PCR

Primer name	Primer sequences	
Mouse METTL3	forward	5′-CTGGGCACTTGGATTTAAGGAA-3′
	reverse	5′-TGAGAGGTGGTGTAGCAACTT-3′
Mouse METTL14	forward	5′-GAGCTGAGAGTGCGGATAGC-3′
	reverse	5′-GCAGATGTATCATAGGAAGCCC-3′
Mouse WTAP	forward	5′-GAACCTCTTCCTAAAAAGGTCCG-3′
	reverse	5′-TTAACTCATCCCGTGCCATAAC-3′
Mouse FTO	forward	5′-GACACTTGGCTTCCTTACCTG-3′
	reverse	5′-CTCACCACGTCCCGAAACAA-3′
Mouse ALKBH5	forward	5′-GCATACGGCCTCAGGACATTA-3′
	reverse	5′-TTCCAATCGCGGTGCATCTAA-3′
Human METTL14	forward	5′-AGTGCCGACAGCATTGGTG-3′
	reverse	5′-GGAGCAGAGGTATCATAGGAAGC-3′
Mouse MCP-1	forward	5′-TAAAAACCTGGATCGGAACCAAA-3′
	reverse	5′-GCATTAGCTTCAGATTTACGGGT-3′
Mouse IL-6	forward	5′-TAGTCCTTCCTACCCCAATTTCC-3′
	reverse	5′-TTGGTCCTTAGCCACTCCTTC-3′
Mouse TNF-α	forward	5′-CCCTCACACTCAGATCATCTTCT-3′
	reverse	5′-GCTACGACGTGGGCTACAG-3′
Human MCP-1	forward	5′-CAGCCAGATGCAATCAATGCC-3′
	reverse	5′-TGGAATCCTGAACCCACTTCT-3′
Human IL-6	forward	5′-CCTTCCAAAGATGGCTGAAA-3′
	reverse	5′-GCTCTGGCTTGTTCCTCACT-3′
Human TNF-α	forward	5′-GAGGCCAAGCCCTGGTATG-3′
	reverse	5′-CGGGCCGATTGATCTCAGC-3′
Human Sirt1	forward	5′-TAGCCTTGTCAGATAAGGAAGGA-3′
	reverse	5′-ACAGCTTCACAGTCAACTTTGT-3′
18S	forward	5′-CGGCTACCACATCCAAGGAA-3′
	reverse	5′-CCTGTATTGTTATTTTTCGTCACTACCT-3′
